# Fractionated robotic radiosurgery for unfavorable nonfunctioning pituitary macroadenoma: 5-year outcomes from a single institution protocol

**DOI:** 10.3389/fonc.2025.1519445

**Published:** 2025-02-04

**Authors:** Akrita Bhatnagar, Monica Pernia Marin, Jonathan W. Lischalk, Min Ji Koh, Siviero Agazzi, Simeng Suy, Brent T. Harris, Susmeeta T. Sharma, Edward Aulisi, Amjad Anaizi, Mohamed H. Khattab, Walter C. Jean, Sean P. Collins, Brian T. Collins

**Affiliations:** ^1^ Department of Radiation Medicine, Georgetown University Hospital, Washington, DC, United States; ^2^ Division of Neuro-Oncology, Columbia University Irving Medical Center, New York, NY, United States; ^3^ Department of Radiation Oncology, Perlmutter Cancer Center, New York University (NYU), Langone, New York, NY, United States; ^4^ Department of Neurosurgery and Brain Repair, University of South Florida, Tampa, FL, United States; ^5^ Department of Pathology and Neurology, Georgetown University Hospital, Washington, DC, United States; ^6^ Department of Endocrinology, MedStar Washington Hospital Center, Washington, DC, United States; ^7^ Department of Neurosurgery, MedStar Washington Hospital Center, Washington, DC, United States; ^8^ Department of Neurosurgery, Georgetown University Hospital, Washington, DC, United States; ^9^ Department of Radiation Oncology, University of South Florida, Tampa, FL, United States; ^10^ Division of Neurosurgery, Lehigh Valley Fleming Neuroscience Institute, Allentown, PA, United States

**Keywords:** radiosurgery, fractionated stereotactic radiosurgery, nonfunctioning pituitary adenoma, local control, CyberKnife

## Abstract

**Objective:**

Nonfunctioning macroadenoma is a commonly diagnosed pituitary tumor. Resection is the favored treatment, with radiosurgery often utilized for residual or progressing disease. Long-term outcomes are established in the literature for single-fraction frame-based radiosurgery, but mature outcomes are lacking for fractionated frameless radiosurgery. We report our institution’s 5-year efficacy and toxicity results for unfavorable nonfunctioning pituitary macroadenoma patients treated with 5-fraction robotic radiosurgery.

**Methods:**

Between 2010 and 2020, patients who completed 5-fraction robotic radiosurgery for the treatment of unfavorable nonfunctioning pituitary macroadenomas were included. A tumor was considered unfavorable if the gross tumor volume (GTV) was larger than 5 cc or if it closely approached a critical structure (optic apparatus, brainstem, or pituitary gland). Local control was calculated using the Kaplan–Meier method.

**Results:**

Twenty predominantly female patients (60%), ages 21–77 (median: 53 years), were included in this study. All underwent primary resection at the time of diagnosis. The indication for radiosurgery was tumor progression (*n* = 14, 70%) or residual tumor after subtotal resection (*n* = 6, 30%). Eighty-five percent of patients treated with radiosurgery (*n* = 17) had cavernous sinus involvement. Median GTV was 3.4 cm^3^ (range: 0.3–20.8 cm^3^), and 40% of the tumors had suprasellar extension. A mean dose of 28.8 Gy (range: 25–30 Gy) was delivered to a median isodose line of 80% (range: 75%–89%). The median optic chiasm maximum point dose was 21.8 Gy (range: 12.0–25.0 Gy). Acute toxicity was minimal with eight patients (40%) developing short-lived headaches and one patient (5%) developing a brief ipsilateral sixth nerve palsy. There was no late radiation-induced neurologic or optic dysfunction identified in this cohort. At a median follow-up of 5 years, local control was 94%. There was one in-field failure pathologically confirmed following surgery for pituitary hemorrhage and two radiographically confirmed out-of-field failures in patients with larger tumors (>20 cc).

**Conclusions:**

The treatment of unfavorable nonfunctioning pituitary macroadenoma with 5-fraction robotic radiosurgery provides excellent local control to date, with acceptable toxicity. However, tumors with GTVs greater than 20 cc may still require conventionally fractionated treatment with a margin to optimize local control.

## Introduction

Pituitary adenomas represent 10%–20% of intracranial tumors and can be hormonally active or non-functioning adenomas (NFAs) (1). Most NFAs are macroadenomas (i.e., measure greater than 1 cm in size at diagnosis) and are diagnosed after developing tumor-related headaches, vision loss, and/or cranial nerve deficit. Some cases are diagnosed incidentally when cranial imaging is performed for other purposes (2).

Multiple treatment options exist for patients with newly diagnosed NFAs, including observation, surgery, or radiation therapy. Transsphenoidal resection is the treatment of choice for patients suitable for surgery. Complete resection is achieved in more than 50% of patients with a low rate of complications including visual field deficits, pituitary dysfunction, cerebrospinal fluid (CSF) rhinorrhea, meningitis, diabetes insipidus, and severe bleeding (3). Unfortunately, complete resection is difficult to accomplish in patients presenting with parasellar extension. Such tumors often require radiation therapy following surgery (3–5).

Radiation therapy is an alternative to resection for patients ill-suited for surgery, an adjuvant treatment for those who have undergone a subtotal resection, or salvage treatment for those who experience tumor recurrence or growth of residual tumor. Multiple radiotherapy modalities are available for the treatment of pituitary macroadenomas, including stereotactic radiosurgery (SRS), multifraction SRS, also known as hypofractionated stereotactic radiotherapy (HSRT), or conventionally fractionated radiation therapy. SRS is a radiation therapy technique that precisely delivers a high dose of radiation in a single fraction while HSRT delivers treatment typically in 2–5 fractions. SRS is often delivered via the frame-based cobalt-60 Gamma Knife (GK) or the image guided CyberKnife (CK) robotic radiosurgery system. HSRT may be delivered using GK, CK, or a modified conventional radiotherapy machine (linear accelerator, LINAC) (6).

SRS is the favored treatment for small pituitary adenomas (<2.5 cm or 5 cc). Multiple retrospective series have demonstrated favorable control rates, often >90% at 5 years and toxicity is low (7). Furthermore, if the pituitary gland can be excluded from the irradiated volume, the risk of radiation-induced hypopituitarism may be negligible with this approach.

However, for unfavorable tumors, i.e., larger tumors, or those in close proximity to critical structures, conventional fractionated radiotherapy uses the advantage of the variance in biologically effective dose to tumors and normal tissue, a difference especially amplified with fractionation, allowing protection of the optic chiasm, proximal optic nerves, pituitary gland, and brainstem when irradiated. This highly effective treatment, with greater than 90% local control at 5 years, has typically been delivered in 25–28 fractions to a total dose of 45–50.4 Gy using a conventional LINAC (8–10).

However, a traditional LINAC has intrafraction uncertainty that necessitates a planning target volume (PTV) expansion, which increases the treatment volume, delivering dose to a larger component of the optic pathway, the pituitary gland, and brain (9, 11). As an alternative, the robotic CK system is a frameless radiosurgical technology with automated intrafraction kV imaging used to assess and automatically adjust to any minimal cranial motion in real time. This technique delivers a conformal radiation dose with a steep dose drop-off like that of the GK and eliminates the need for a margin accounting for intrafraction uncertainty. Additionally, because of the precise intrafraction positional correction, patients do not require frame-based immobilization, allowing for enhanced patient comfort (12–14).

A recent large meta-analysis confirmed that with single-fraction SRS, approximately 8% of patients developed vision loss and approximately 20% of all patients developed hypopituitarism. The authors concluded that multifraction SRS delivered in 3 to 5 fractions is promising with high rates of local control and low rates of treatment-related toxicity. However, they acknowledged that mature outcomes are lacking for multisession radiosurgery and therefore do not yet recommend it for routine clinical practice (7). To our knowledge, only two studies thus far have specifically looked at HSRT for non-functioning pituitary adenomas (15, 16).

Of these studies, one study reported outcomes using a NovalisTX radiotherapy system and the other utilized CK. Khattab and colleagues, treating on a NovalisTX radiotherapy system, reported their outcomes with HSRT for large nonfunctioning pituitary adenomas with chiasm involvement showing over 92% local control in the cohort treated with HSRT and with no optic neuropathies and similar endocrinopathies to the single-fraction cohort (15). Iwata and colleagues reported their outcomes on a CK treating 100 patients with NFPAs with HSRT with 98% local control and very low toxicity, with only 1 patient having grade 2 visual symptoms at a 3-year follow-up (16). In this small retrospective study, we report our promising 5-year efficacy and toxicity results for unfavorable NFPA patients treated with 5-fraction robotic radiosurgery.

## Materials and methods

### Study design, setting, and participants

The Medstar Health Research Institute–Georgetown University Oncology institutional review board approved this retrospective analysis of an established departmental treatment approach. A multidisciplinary neuro-oncology team evaluated patients. Patients with unfavorable nonfunctioning pituitary macroadenomas who had undergone surgical resection followed by 5-fraction robotic radiosurgery from 2010 to 2020 were included in the present analysis. A tumor was considered unfavorable if the gross tumor volume (GTV) was greater than 5 cc or if the tumor closely approached a critical structure (e.g., optic apparatus, pituitary gland, or brainstem).

### Treatment planning

Prior to treatment, a custom thermoplastic mask was fabricated. A fine-cut (1.25 mm) contrast-enhanced treatment planning CT scan was obtained in the supine treatment position for each patient using a GE LightSpeed RT16. T1-weighted, contrast-enhanced magnetic resonance imaging (MRI) with 1.25-mm slice thickness was fused with the planning CT scans for target volume delineation. Pituitary adenomas and organs at risk (i.e., optic nerve, optic chiasm, and brainstem) were contoured without expansion on all visualized image slices of the planning CT scan and/or fused MRI. The pituitary gland was contoured as an organ at risk when identified. A treatment plan was generated using the Accuray Inc. MultiPlan 5.2.1 non-isocentric inverse-planning algorithm.

### Delivery of stereotactic fractionated radiotherapy

Each patient underwent fractionated CK radiosurgery (Accuray Inc.) (17). Treatment plans were composed of hundreds of pencil beams delivered using a single 10- to 35-mm-diameter collimator. Radiation was delivered in 5 fractions of 5 to 6 Gy prescribed to an isodose line that covered at least 95% of the GTV. Patients were treated in the supine position with a custom aquaplast mask for immobilization and reproducible patient setup. The SRS treatment was routinely delivered over five consecutive business days. Organ at risk *D*_max_ constraints were defined as 25 Gy for the optic chiasm, 28 Gy for optic nerves, and 31 Gy for the brainstem. There was no dose constraint placed on the pituitary gland.

### Follow-up studies

Patients were followed with serial physical examination, pituitary function tests, and MRI per routine institutional practice. Routine visual field testing was not completed. Local tumor recurrence was defined as progression of the treated tumor based on radiological review of serial follow-up MRI. All cases involving a question of tumor progression were discussed at a weekly interdisciplinary CNS tumor conference. Progression was confirmed pathologically in those cases requiring surgical intervention. Toxicities were scored according to the National Cancer Institute Common Terminology Criteria for Adverse Events, Version 5.0 (18). A patient was considered to have radiation-induced pituitary dysfunction if they developed a new hormone deficiency requiring medical treatment following radiosurgery. A patient was considered to have radiation-induced vision loss if they developed a new visual field deficit following radiosurgery confirmed by formal visual field testing. Patients were followed until death. Cause of death analysis was completed by the interdisciplinary CNS tumor conference. Autopsy was not completed to confirm cause of death.

### Statistical analysis

Data were analyzed and graphs were prepared with the SPSS 23 statistical package (IBM Corporation, Armonk, NY). The follow-up duration was defined as the time from the date of completion of radiosurgery treatment to the last date of follow-up or the date of death. The treatment parameters assessed included treatment isodose line, tumor coverage, and maximum radiation dose to organs at risk. The conformity index (CI) is the ratio of the treatment volume to the target volume.

The primary endpoint was local control defined as stable disease or partial response as evaluated on serial MRI. These were calculated using the Kaplan–Meier method. The secondary endpoints were radiation-induced neurologic or optic dysfunction.

## Results

### Patient, tumor, and treatment characteristics

Twenty predominantly female patients (60%), ages 21–77 (median: 53 years), were included in this study (**Table 1** **).** All underwent primary resection. One patient had panhypopituitarism, and two patients had visual field deficits prior to radiosurgery. The indication for radiosurgery was tumor progression in 70% of patients. The remainder were treated following subtotal resection (30%). Eighty-five percent of patients treated with radiosurgery had been deemed unresectable due to cavernous sinus involvement. Pituitary gland contouring for radiosurgery planning was feasible in eight patients (40%). The median tumor volume was 3.4 cc (range: 0.3–20.8 cc). Thirty percent of the tumors were greater than 5.00 cc and 40% of the tumors were suprasellar (**Table 2**).

**Table 1:** Patient characteristics.

Age	
Median	53
Range	21–77

Sex, *n* (%)	
Male	8 (40)
Female	12 (60)

Race, *n* (%)	
Caucasian	9 (45)
African American	9 (45)
Hispanic or Latino	2 (10)

ECOG, *n* (%)	
0–1	35 (70)
2–3	15 (30)

**Table 2:** Tumor characteristics.

Indications for radiosurgery, *n* (%)	
Subtotal resection	6 (30)
Tumor progression	14 (70)

Tumor extension, %	
Suprasellar	8 (40)
Cavernous sinus	17 (85)

Gross tumor volume, cc	
Median	3.4
Range	(0.3–20.8)
Gross tumor volume < 5 cc, *n* (%)	14 (70)
Gross tumor volume > 5 cc but <20 cc, *n* (%)	4 (20)
Gross tumor volume > 20 cc, *n* (%)	2 (10)

### Stereotactic fractionated radiosurgery

A mean dose of 28.8 Gy (range: 25–30 Gy) was delivered to a median isodose line of 80% (range: 75%–89%). The median tumor volume (GTV) treated was 3.8 cc (range: 0.3–20.8 cc). The mean GTV target coverage was 99.88% (range: 97%–100%) and average conformality index was of 1.63. The median optic nerve and optic chiasm maximum point doses were 22.9 Gy (range: 10.8–27.9 Gy) and 21.8 Gy (range: 12.0–25.0 Gy). The median pituitary gland maximum point dose was 30.5 Gy (range: 23.8–35.1 Gy) and mean dose was 20.2 Gy (range: 14.6–29.5 Gy) (**Table 3**).

**Table 3:** Treatment dose and maximum point dose of organs at risk.

Patient ID	Total dose (cGy)	Isodose line (%)	Optic chiasm *D*_max_ (Gy)	Optic nerve *D*_max_ (Gy)	Brainstem *D*_max_ (Gy)	Pituitary *D*_max_ (Gy)	Pituitary *D*_mean_ (Gy)
Patient 1	2,500	86	25.00	26.12	13.16	Unknown*	Unknown*
Patient 2	2,500	75	24.97	22.32	23.51	Unknown*	Unknown*
Patient 3	2,500	80	22.38	21.57	23.76	Unknown*	Unknown*
Patient 4	2,500	82	22.88	27.92	28.25	Unknown*	Unknown*
Patient 5	3,000	82	11.99	20.84	29.48	Unknown*	Unknown*
Patient 6	3,000	80	15.37	21.07	25.91	32.85	16.98
Patient 7	3,000	75	25.00	24.54	12.68	30.97	22.42
Patient 8	3,000	84	23.34	25.91	8.39	31.28	26.35
Patient 9	2,500	83	24.42	22.19	12.55	Unknown*	Unknown*
Patient 10	2,500	89	21.35	21.38	9.01	Unknown*	Unknown*
Patient 11	2,500	87	18.87	18.25	18.62	Unknown*	Unknown*
Patient 12	2,500	83	13.50	23.71	25.22	Unknown*	Unknown*
Patient 13	2,500	78	13.50	23.71	25.22	Unknown*	Unknown*
Patient 14	3,000	88	21.58	24.82	18.56	23.76	14.57
Patient 15	3,000	80	24.12	24.72	22.16	35.11	29.54
Patient 16	3,000	80	20.66	21.83	9.04	Unknown*	Unknown*
Patient 17	2,500	83	21.32	21.37	16.35	Unknown*	Unknown*
Patient 18	3,000	77	15.23	10.75	7.61	31.86	18.76
Patient 19	3,000	86	21.98	26.96	23.99	24.29	15.92
Patient 20	3,000	80	22.74	23.54	26.96	34.10	21.58

*The pituitary gland was not always visible for contouring.

### Tumor control and survival

**Figure 1:**
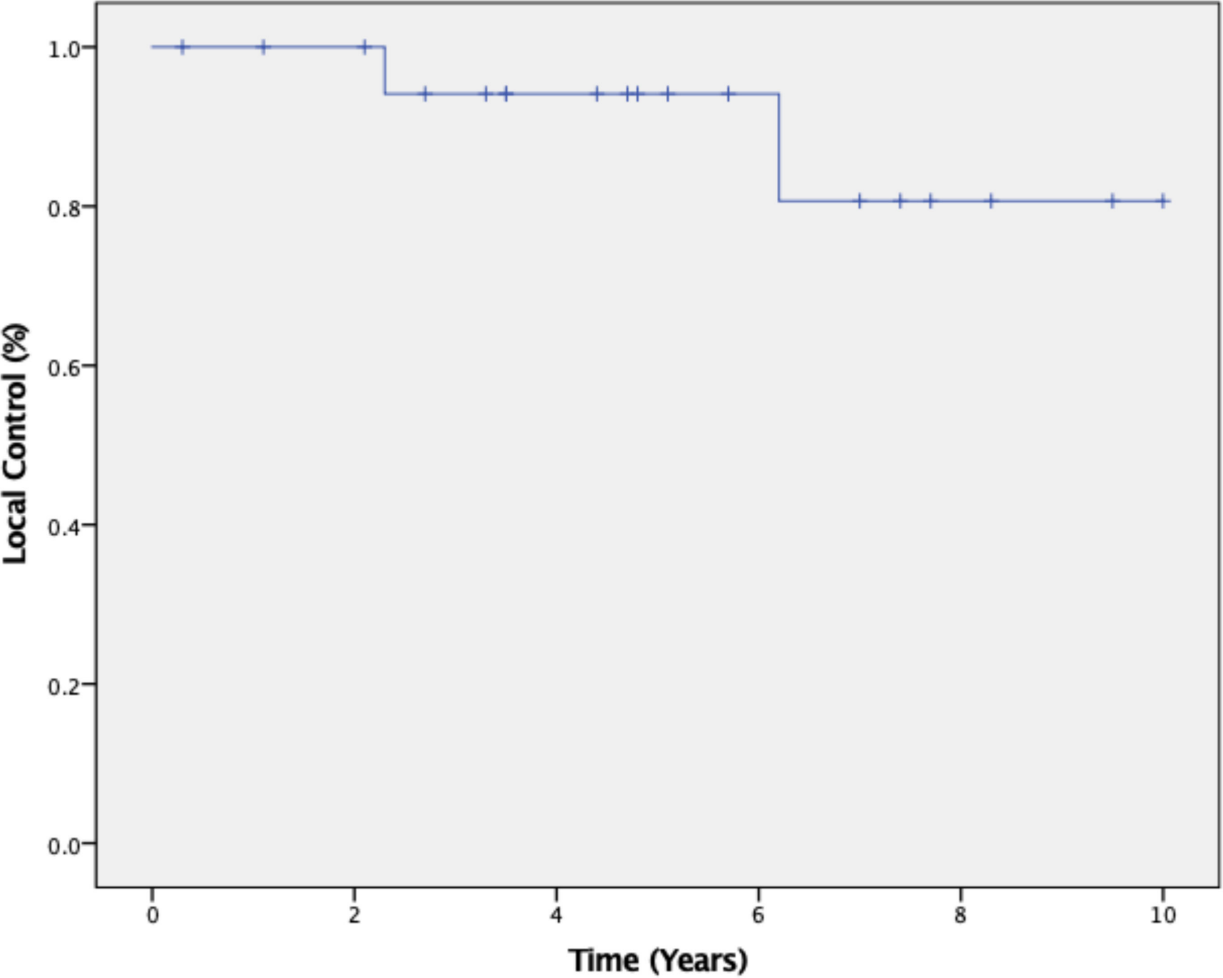
Kaplan–Meier 5-year local control was 94% at a median follow-up of 5 years from completion of SRS radiotherapy treatment.

**Figure 2:**
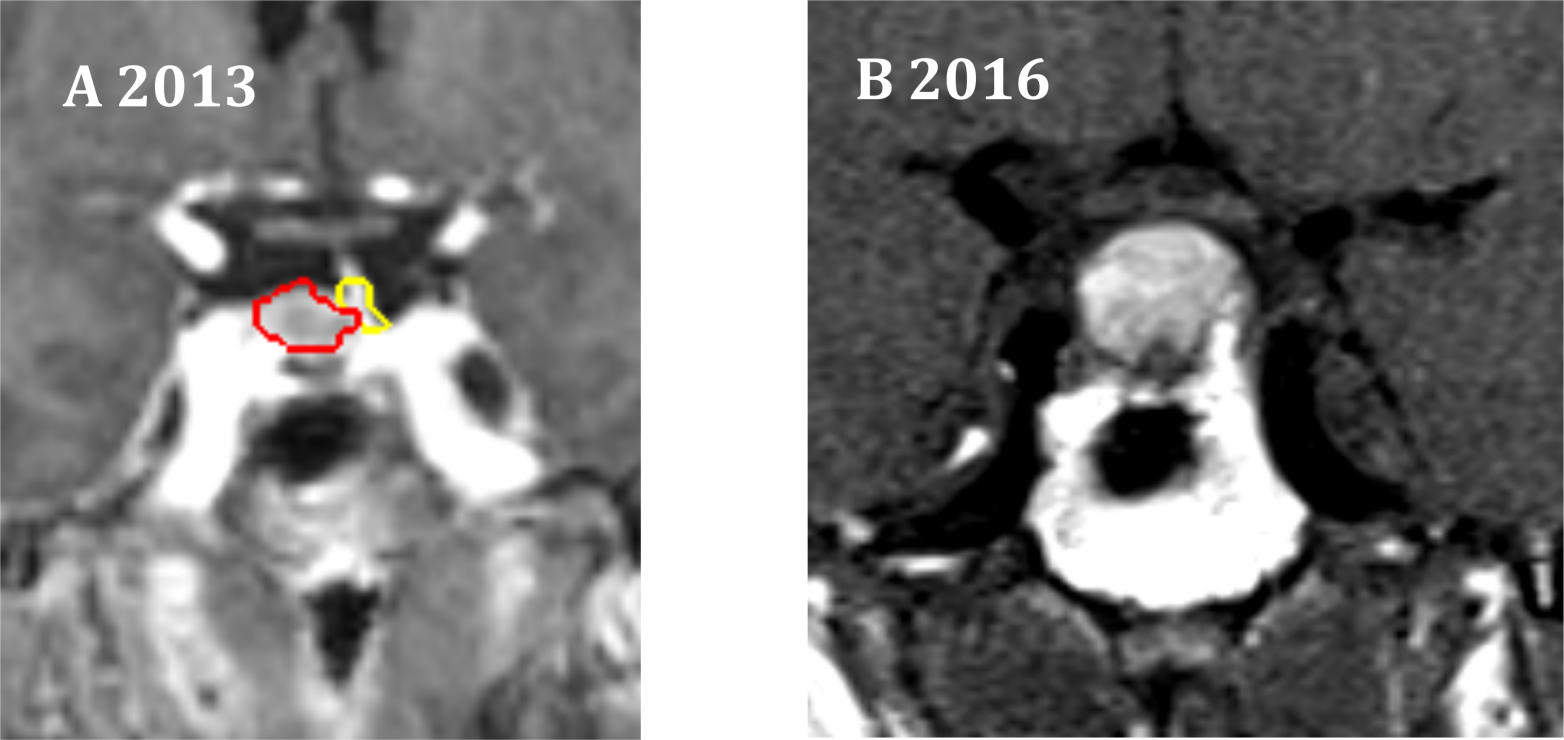
One in-field failure. **(A)** Coronal radiation treatment image demonstrating the treatment volumes for an NFA. Red represents the gross tumor volume (GTV), which is 1 cc in size. Yellow represents the pituitary gland. A prescription dose of 30 Gy was delivered to the 83% isodose line. **(B)** At 3 years, pituitary hemorrhage was observed.

**Figure 3:**
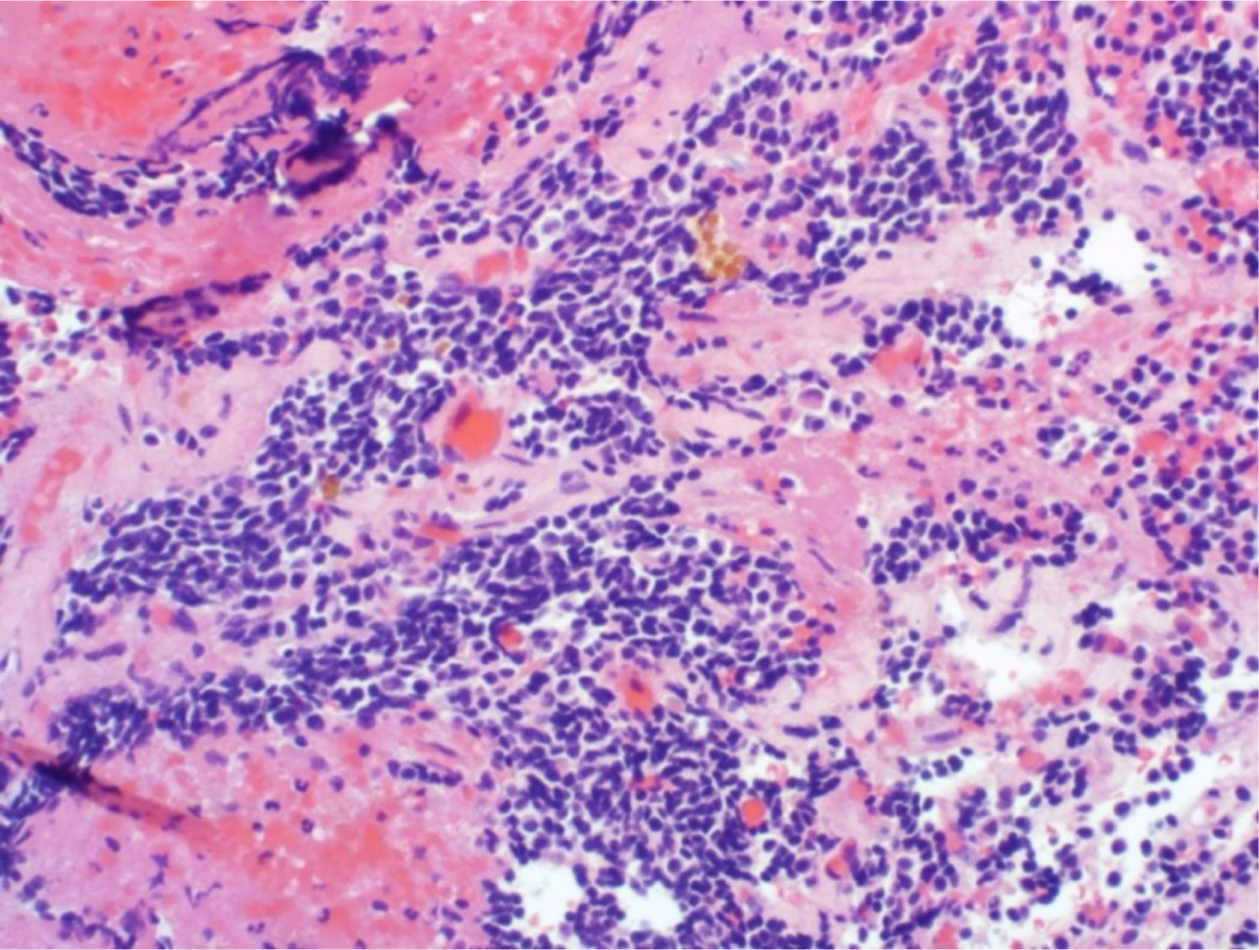
There was one early in-field failure pathologically confirmed following surgery for pituitary hemorrhage. Microscopic evaluation revealed residual pituitary adenoma with fibrosis and hemorrhage.

**Figure 4:**
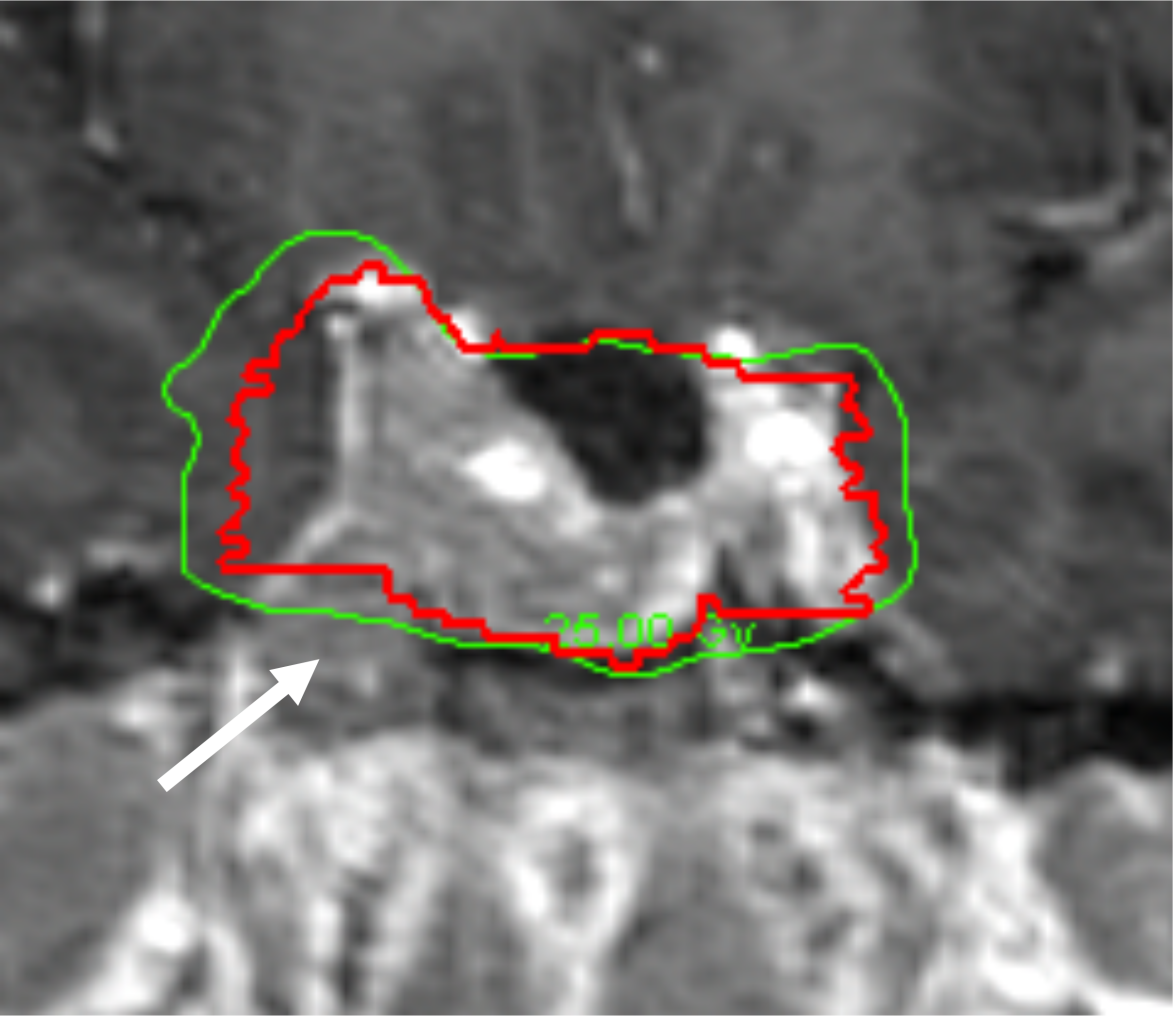
Example of a radiographically confirmed out-of-field failure in a patient with a large tumor (>20 cc) treated with a lower dose (25 Gy). Red: gross tumor volume (GTV) at the time of treatment. Green: isodose line that indicates the prescribed dose of 25 Gy. See tumor progression inferior to the prescribed dose of 25 Gy (white arrow).

The 5-year tumor control rate was 94% at a median follow-up of 5 years (**Figure 1**). There were three local failures (**Table 4**). One was an early in-field failure for a 1-cc tumor that received a dose of 30 Gy (**Figure 2**). It was pathologically confirmed following surgery for pituitary hemorrhage (**Figure 3**). The other two local failures were late radiographically confirmed out-of-field failures in patients with large tumors (>20 cc) that had received a relatively low dose of 25 Gy (**Figure 4**). There were two reported deaths in this study that were unrelated to the pituitary tumor or its treatment.

**Table 4:** Tumor characteristics and fSRS outcomes.

Patient ID	Presentation	Approach	Cavernous sinus extension	Suprasellar extension	Unfavorable tumor characteristic compelling fractionation	Local failure	Acute toxicity	Radiation-induced pituitary dysfunction
Patient 1	Vision loss	Salvage	Yes	Yes	Optic apparatus proximity	No	Headache	Yes
Patient 2	Incidental	Adjuvant	Yes	Yes	Optic apparatus proximity	Yes	None	No
Patient 3	Vision loss	Adjuvant	Yes	No	Gross tumor vol > 5 cc	Yes	Headache	No
Patient 4	Vision loss	Salvage	Yes	Yes	Gross tumor vol > 5 cc	No	Headache	No
Patient 5	Amenorrhea	Salvage	Yes	No	Brainstem proximity	No	Headache	No
Patient 6	Libido	Salvage	Yes	No	Gross tumor vol > 5 cc	No	Cranial nerve VI deficit	No
Patient 7	Vision loss	Salvage	No	Yes	Optic apparatus proximity	No	None	No
Patient 8	Vision loss	Salvage	Yes	Yes	Optic apparatus proximity	Yes	None	N/A
Patient 9	Vision loss	Adjuvant	No	Yes	Optic apparatus proximity	No	None	No
Patient 10	Incidental	Adjuvant	Yes	No	Optic apparatus proximity	No	None	No
Patient 11	Headache	Salvage	Yes	No	Gross tumor vol > 5 cc	No	None	No
Patient 12	Vision loss	Salvage	Yes	No	Optic apparatus proximity	No	None	No
Patient 13	Vision loss	Adjuvant	Yes	No	Gross tumor vol > 5 cc	No	Headache	No
Patient 14	Vision loss	Salvage	Yes	No	Optic apparatus proximity	No	None	No
Patient 15	Galactorrhea	Salvage	Yes	Yes	Pituitary gland proximity	No	Headache	Yes
Patient 16	Amenorrhea	Salvage	Yes	No	Optic apparatus proximity	No	Headache	No
Patient 17	Vision loss	Salvage	Yes	No	Gross tumor vol > 5 cc	No	None	No
Patient 18	Vision loss	Salvage	Yes	No	Pituitary gland proximity	No	Headache	No
Patient 19	Vision loss	Salvage	Yes	No	Optic apparatus proximity	No	None	No
Patient 20	Cavernous sinus syndrome	Adjuvant	No	Yes	Pituitary gland proximity	No	None	N/A

### Toxicity

Toxicity was acceptable with eight patients (40%) developing acute transient headaches and one patient (5%) developing an acute brief ipsilateral sixth nerve palsy (**Table 4**) Two patients developed chronic hypothyroidism, approximately 4 years after the completion of radiation treatment that required hormone replacement therapy (**Table 4**). The pituitary gland had been contoured in one of these patients and had received high maximum and mean pituitary doses of 31.28 and 26.35 Gy.

## Discussion

Single-fraction SRS delivering doses of 14–16 Gy is a standard treatment option for small (<2.5 cm) residual, recurrent, or progressing NFAs, resulting in tumor control rates of 95% at 5 years and acceptable toxicity (i.e., ~20% chronic pituitary dysfunction) (7, 14). Our results suggest that SRS delivered in 5 fractions to a total dose of 25–30 Gy may represent an alternative treatment option for unfavorable nonfunctioning pituitary macroadenoma with similar 5-year local control rates.

Our reported toxicities were minimal. Multifraction SRS schemes allow for a high dose of conformal radiation to be delivered to a pituitary tumor with a steep dose fall-off. The pituitary gland was often in close proximity to the tumor and at times was not discernable. In this situation, limited fractionation allowed us to protect organs at risk including the pituitary gland. Hypopituitarism remains the most commonly reported late complication following pituitary tumor radiation treatment (7). In our routine radiosurgery practice, we have now adopted a 5-fraction treatment approach for all cases of pituitary macroadenomas to minimize radiation toxicity.

We observed one in-field failure for a 1-cc tumor that received a total dose of 30 Gy (**Figure 2**). It was pathologically confirmed following surgery for pituitary hemorrhage (**Figure 3**). Pituitary hemorrhage following single-fraction radiosurgery is not an uncommon phenomenon with a reported incidence of 7% (19–21). We do not believe that this toxicity was a result of fractionation. In fact, pituitary hemorrhage might also be decreased with fractionation. Only one patient experienced pituitary hemorrhage in our small series (i.e., 5% of patients). There were two radiographically confirmed out-of-field failures in patients with large tumors (>20 cc) treated with lower doses (25 Gy) (**Table 3**). See tumor progression inferior to the prescribed dose (**Figure 4**). Therefore, we believe tumors with GTVs greater than 20 cc may still require conventionally fractionated treatment with a margin to optimize local control (22).

Limitations of this study include the small patient population and its retrospective design. Because of its retrospective design, we recognize that we did not have baseline formal visual field testing or a comprehensive pituitary function evaluation, prior to and after treatment. Thus, our toxicity could be more significant than reported. Future prospective research should continue to investigate the optimal fractionation schedule for non-functioning pituitary macroadenoma.

## Conclusion

Single-fraction radiosurgery is the favored treatment for small (<2.5 cm or 5 cc) residual, recurrent, or progressing NFAs. However, a recent large meta-analysis suggests that for tumors that are >2.5 cm or 5 cc, or located in close proximity to the optic apparatus, SRS delivered in 3 to 5 fractions may represent an alternative treatment to single-fraction SRS. However, the absence of mature outcome data has prevented this from becoming a routine approach (7). We present the first 5-year outcome data in a small series of patients that confirm that the treatment of unfavorable nonfunctioning pituitary macroadenoma with 5-fraction CK radiosurgery provides excellent local control, 94% at 5 years, with minimal acute toxicity and no late radiation-induced neurologic or optic deficits. However, our small series also suggests that tumors with GTVs greater than 20 cc may still require conventionally fractionated treatment with a margin to optimize local control. Prospective research is planned to confirm our preliminary findings.

## Data Availability

The raw data supporting the conclusions of this article will be made available by the authors, without undue reservation.

## References

[1] Ezzat S Asa SL Couldwell WT Barr CE Dodge WE Vance ML. The prevalence of pituitary adenomas: a systematic review. Cancer. (2004) 101:613–9. doi: 10.1002/cncr.20412 15274075

[2] Drummond JB Ribeiro-Oliveira A Jr. Soares BS. Non-Functioning Pituitary Adenomas. In: Feingold KR Anawalt B Blackman MR, editors. Endotext. MDText.com, Inc (2000). Copyright ^©^ 2000-2024. Available at: https://www.ncbi.nlm.nih.gov/books/NBK534880/.

[3] Mortini P Losa M Barzaghi R Boari N Giovanelli M. Results of transsphenoidal surgery in a large series of patients with pituitary adenoma. Neurosurgery. (2005) 56:1222–33. doi: 10.1227/01.neu.0000159647.64275.9d 15918938

[4] Esposito D Olsson DS Ragnarsson O Buchfelder M Skoglund T Johannsson G. Non-functioning pituitary adenomas: indications for pituitary surgery and post-surgical management. Pituitary. (2019) 22:422–34. doi: 10.1007/s11102-019-00960-0 PMC6647426 31011999

[5] Dekkers OM Pereira AM Romijn JA. Treatment and follow-up of clinically nonfunctioning pituitary macroadenomas. J Clin Endocrinol Metab. (2008) 93:3717–26. doi: 10.1210/jc.2008-0643 18682516

[6] Minniti G Osti MF Niyazi M. Target delineation and optimal radiosurgical dose for pituitary tumors. Radiat Oncol. (2016) 11:135. doi: 10.1186/s13014-016-0710-y 27729088 PMC5057503

[7] Kotecha R Sahgal A Rubens M Salles AD Fariselli L Pollock BE. Stereotactic radiosurgery for non-functioning pituitary adenomas: meta-analysis and International Stereotactic Radiosurgery Society practice opinion. Neuro Oncol. (2020) 22:318–32. doi: 10.1093/neuonc/noz225 PMC7058447 31790121

[8] Purdy JA. Dose to normal tissues outside the radiation therapy patient’s treated volume: a review of different radiation therapy techniques. Health Phys. (2008) 95:666–76. doi: 10.1097/01.Hp.0000326342.47348.06 18849701

[9] Milker-Zabel S Debus J Thilmann C Schlegel W Wannenmacher M. Fractionated stereotactically guided radiotherapy and radiosurgery in the treatment of functional and nonfunctional adenomas of the pituitary gland. Int J Radiat Oncol Biol Phys. (2001) 50:1279–86. doi: 10.1016/s0360-3016(01)01535-8 11483339

[10] Kong DS Lee JI Lim DH Kim KW Shin HJ Nam D-H. The efficacy of fractionated radiotherapy and stereotactic radiosurgery for pituitary adenomas: long-term results of 125 consecutive patients treated in a single institution. Cancer. (2007) 110:854–60. doi: 10.1002/cncr.22860 17599761

[11] Leber KA Berglöff J Pendl G. Dose-response tolerance of the visual pathways and cranial nerves of the cavernous sinus to stereotactic radiosurgery. J Neurosurg. (1998) 88:43–50. doi: 10.3171/jns.1998.88.1.0043 9420071

[12] Chang SD Main W Martin DP Gibbs IC Heilbrun MP. An analysis of the accuracy of the CyberKnife: a robotic frameless stereotactic radiosurgical system. Neurosurgery. (2003) 52:140–6. doi: 10.1097/00006123-200301000-00018 12493111

[13] Killory BD Kresl JJ Wait SD Ponce FA Porter R White WL. Hypofractionated CyberKnife radiosurgery for perichiasmatic pituitary adenomas: early results. Neurosurgery. (2009) 64:A19–25. doi: 10.1227/01.Neu.0000341630.42160.18 19165069

[14] Adler JR Jr. Gibbs IC Puataweepong P Chang SD. Visual field preservation after multisession cyberknife radiosurgery for perioptic lesions. Neurosurgery. (2006) 59:244–54. doi: 10.1227/01.Neu.0000223512.09115.3e 16883165

[15] Khattab MH Sherry AD Xu MC Kelly P Anderson JL Luo G. Stereotactic radiosurgery and hypofractionated stereotactic radiotherapy for nonfunctioning pituitary adenoma. J Neurol Surg B Skull Base. (2021) 82:e51–8. doi: 10.1055/s-0040-1710518 PMC8289501 34306917

[16] Iwata H Sato K Tatewaki K Yokota N Inoue M Baba Y. Hypofractionated stereotactic radiotherapy with CyberKnife for nonfunctioning pituitary adenoma: high local control with low toxicity. Neuro Oncol. (2011) 13:916–22. doi: 10.1093/neuonc/nor055 PMC3145469 21665918

[17] Heilbrun MP. Cyberknife Radiosurgery: A Practical Guide. Sunnyvale, CA: CyberKnife Society (2003).

[18] Institute NC. Common Terminology Criteria for Adverse Events (CTCAE). National Cancer Institute. Available online at: https://ctep.cancer.gov/protocoldevelopment/electronic_applications/docs/CTCAE_v5_Quick_Reference_5x7.pdf (Accessed October 29, 2024).

[19] Fu J Li Y Wu L Yang X Quan T Li X. Pituitary hemorrhage in pituitary adenomas treated with gamma knife radiosurgery: incidence, risk factors and prognosis. J Cancer. (2021) 12:1365–72. doi: 10.7150/jca.52349 PMC7847656 33531981

[20] Weisberg LA. Pituitary apoplexy. Association of degenerative change in pituitary ademona with radiotherapy and detection by cerebral computed tomography. Am J Med. (1977) 63:109–15. doi: 10.1016/0002-9343(77)90122-x 879188

[21] Briet C Salenave S Bonneville JF Laws ER Chanson P. Pituitary apoplexy. Endocr Rev. (2015) 36:622–45. doi: 10.1210/er.2015-1042 26414232

[22] Minniti G Flickinger J Tolu B Paolini S. Management of nonfunctioning pituitary tumors: radiotherapy. Pituitary. (2018) 21:154–61. doi: 10.1007/s11102-018-0868-4 29372392

